# Exploring cognitive and motivational impacts of flipped English classrooms: perspectives from teachers in a Chinese art university

**DOI:** 10.3389/fpsyg.2025.1647363

**Published:** 2026-01-12

**Authors:** Yingxue Ling, Jariah Mohd Jan

**Affiliations:** 1 Department of Foreign Studies, Yili Normal University, Yining, China; 2 Department of English Language, Faculty of Languages and Linguistics, Universiti Malaya, Kuala Lumpur, Malaysia

**Keywords:** Chinese art university, cognitive impact, flipped classroom, motivational impact, teacher experience

## Abstract

**Introduction:**

This qualitative study examines the cognitive and motivational impacts of flipped classrooms on English teachers at a Chinese art university, drawing on self-determination theory and social constructivism.

**Methods:**

Data were collected through semi-structured interviews and classroom observations with English teachers implementing flipped classroom pedagogy, and analyzed thematically to capture teachers’ experiences and perceptions.

**Results:**

The findings indicate that flipped classrooms enhanced teachers’ autonomy, professional satisfaction, and classroom flexibility, facilitating a shift from textbook-based instruction to more student-centered approaches. However, teachers also reported challenges, including student resistance rooted in passive learning habits and the increased workload associated with preparing pre-class materials. These challenges were intensified by contextual constraints in Chinese art education, such as inconsistent access to learning management systems, limited instructional technology support, and insufficient professional development in digital pedagogy.

**Discussion:**

The study highlights the importance of addressing both cognitive and motivational dimensions when implementing flipped classrooms, particularly in resource-constrained and discipline-specific contexts. By foregrounding teachers’ experiences in a specialized institutional setting, this research contributes to flipped learning scholarship and offers practical insights for fostering sustainable pedagogical innovation.

## 1 Introduction

The significance of student-centered learning approaches has been widely recognized in educational research (Freeman et al., 2014). This paradigm shift is driven by technological advancements that reshape not only learning environments but also pedagogical roles and practices (Chen and Tsai, 2021). Teachers are transitioning from traditional knowledge transmitters to facilitators who guide students in accessing and constructing knowledge, while students assume greater responsibility for their learning through active engagement and collaboration (Bishop and Verleger, 2013).

Among emerging student-centered methods, the flipped classroom has gained attention as an innovative instructional model. It reverses the conventional order of instruction by assigning core content for out-of-class study through videos, readings, or other multimedia, thereby freeing classroom time for active learning activities such as discussions, problem-solving, and collaborative projects (Bergmann and Sams, 2014).

Research has demonstrated that flipped classrooms can enhance both cognitive and motivational aspects of learning. Cognitive benefits include improved knowledge acquisition, higher-order thinking skills, and active engagement with course material, all of which contribute to better academic outcomes (Asef-Vaziri, 2015; Kurt, 2017). On the motivational side, flipped learning has been associated with greater learner autonomy, sustained engagement, and increased satisfaction with the learning process (Hung, 2014; Unal and Unal, 2017; Goedhart et al., 2019; Tsai, 2021). However, challenges remain. Students often struggle with the cognitive demands of processing new content independently outside the classroom, and some experience difficulty engaging in higher order thinking without immediate teacher support (Paul et al., 2019). Motivationally, learners may resist the increased responsibility required in self-directed learning, particularly when they are accustomed to passive, teacher-centered instruction (Jakobsen and Knetemann, 2017). These issues highlight the importance of contextual factors in shaping the success of flipped classrooms.

In the Chinese higher education context, national educational reforms have increasingly promoted student-centered pedagogies, including the adoption of flipped classrooms, particularly in English language instruction (Ministry of Education of the People’s Republic of China, 2016; Wang and Zhang, 2013). These reforms aim at broader goals to cultivate learners’ communicative competence, critical thinking, and autonomous learning abilities (Ministry of Education of the People’s Republic of China, 2016). However, despite policy encouragement, the transition to flipped learning models remains uneven. Traditional teacher-centered methods still dominate many classrooms, especially in English courses for non-English majors, where exam-oriented teaching, large class sizes, and rigid curricula often limit opportunities for active learning (Cui and Wang, 2014; Zhao, 2022). Moreover, flipped classroom implementation in China frequently faces challenges such as students’ unfamiliarity with self-directed learning, limited digital pedagogy training of teachers, and institutional constraints (Lu, 2016).

These barriers are further intensified in more specialized settings, such as art universities, where English instruction is often marginalized, and students may have lower motivation, proficiency, and confidence in language learning (Xia, 2012; Li, 2019). Students in these contexts frequently exhibit limited language proficiency, weak academic learning habits, and minimal experience with self-directed study (Li, 2019)—factors that can complicate the adoption of flipped learning models. While these students may struggle with formal language learning, their creativity, openness to multimedia, and preference for experiential learning may align well with the multimodal nature of flipped instruction. As state by Zhang (2012), the inherent creativity, visual orientation, and exploratory learning styles of art students may also offer unique opportunities for implementing active, student-centered pedagogies.

Flipped classrooms, with their emphasis on autonomy, collaboration, and multimodal learning, may better align with these learners’ preferences and potentially support deeper engagement and skill development (Chiu and Hwang, 2024). Despite this potential, empirical research on the effectiveness and practical challenges of flipped classrooms in art university contexts remains scarce (Xia, 2012; Chiu and Hwang, 2024).

Most research is situated in comprehensive universities and tends to emphasize theoretical benefits (Li et al., 2020), general student outcomes (Hu and Wu, 2014; Peng and Cai, 2022), implementation challenges (Chen, 2019), and overall feasibility (Yuan et al., 2022). Although informative, this body of work tends to adopt a broad perspective and frequently overlooks the nuanced cognitive, motivational and interpersonal processes within flipped learning environments.

Recent studies have begun to highlight the importance of psychological and relational factors, showing that teacher–student interactions and perceived support significantly influence student motivation and engagement in flipped learning contexts (Li, 2022; Zhang and Hu, 2023). However, these findings predominantly reflect students’ perspectives, with comparatively less research exploring teachers’ views on these relational dynamics. Instructors are key to the success of flipped classrooms, as they are responsible for organizing learning activities inside and outside the classroom, preparing instructional content, and supporting students throughout the process.

Implementing flipped classroom also involves effectively incorporating digital tools into teaching practices (Davies et al., 2013). For example, Kazu and Kuvvetli (2025) investigated the impact of Duolingo on listening, speaking, reading, and writing skills, showing that digital tools can differentially affect skill development and learner motivation. In addition to language education, technology-enhanced flipped classrooms have been implemented in STEM and other disciplines, demonstrating the generalisability of flipped pedagogy across diverse contexts. For example, flipped classrooms in mathematics have incorporated computer algebra systems to support pre-class learning and in-class problem-solving (Karjanto and Simon, 2019). This broader literature illustrates how digital tools can facilitate active, student-centered learning beyond language courses, providing a comparative backdrop for examining flipped instruction in a Chinese art university. Their findings indicate that technology-mediated language learning can influence both cognitive and motivational outcomes. Situating the present study within this broader landscape allows for comparison between flipped classrooms and other technology-enhanced approaches.

In light of this, the present study explores the perspectives of English teachers implementing flipped classrooms at a Chinese art university. Specifically, it focuses on the cognitive and motivational dynamics experienced by instructors. The study is guided by the following research questions:

*RQ1*: How do Chinese art university teachers perceive the cognitive and motivational impacts of flipped English classrooms on their own teaching experience?

*RQ2*: What cognitive and motivational challenges Chinese art university teachers experienced in the implementation of flipped classrooms in this setting?

## 2 Theoretical framework

To explore Chinese art university teachers’ perceptions of flipped English classrooms, this study draws on two complementary theoretical frameworks: Constructivist Learning Theory (Vygotsky, 1978) and self-determination theory (SDT) (Deci and Ryan, 1985). These frameworks are particularly suited to examining the cognitive and motivational dimensions of teachers’ experiences, as reflected in the study’s three research questions.

Constructivist Learning Theory views learning as an active, social, and reflective process in which individuals construct knowledge through interaction with their environment and others (Vygotsky, 1978). Traditionally applied to student learning, this framework is also relevant for understanding teachers’ cognitive development as they adapt to new pedagogical models. In the context of flipped classrooms—where content delivery is moved outside the classroom and in-class time is reserved for active, collaborative learning—teachers must reconsider their instructional roles, pedagogical strategies, and classroom dynamics. Constructivism provides a lens through which to analyze how teachers reflect on and reconstruct their teaching practices, helping to address the cognitive impacts and challenge.

To investigate the motivational aspects of teachers’ experiences, this study draws on self-determination theory, which posits that motivation is influenced by the fulfillment of three basic psychological needs: autonomy (the need to feel in control), competence (the need to feel effective), and relatedness (the need to feel connected to others) (Deci and Ryan, 1985). In this study, SDT is applied primarily as an interpretive framework rather than a prescriptive design tool. The interview protocol was intentionally kept open-ended to capture a wide range of teacher experiences, rather than being narrowly structured around predefined SDT categories. This exploratory approach allowed unanticipated themes to surface but limited the extent to which motivational constructs were systematically probed at the data collection stage.

## 3 Methodology

This study employed a qualitative single-case study design to explore the experiences of English teachers implementing flipped classrooms at a Chinese art university. A qualitative approach was chosen to gain a deep, contextual understanding of how teachers interpret and respond to this pedagogical shift in their natural teaching environments (Creswell, 2013). Unlike quantitative methods, which require pre-defined constructs and closed-ended instruments, qualitative interviews allow participants to articulate their lived experiences, describe the nuances of their teaching practices, and express context-specific interpretations. The case involved a bounded system (Yin, 2014)—six English instructors engaged in flipped teaching during the 2023–2024 academic year at an art university in Chengdu, Sichuan Province, China. The university is equipped with smart classrooms that support interactive instructional models, including flipped learning.

Participants were selected through purposive sampling to ensure rich, relevant insights. All six teachers had at least 5 years of teaching experience and prior participation in flipped classroom programs. Before implementation, they received professional training on flipped classroom design and instructional strategies, including workshops on lesson planning, multimedia content creation, and techniques for fostering student engagement. They were recommended by the Director of the English Department and selected based on availability and willingness to contribute. Ethical approval was obtained, and all participants gave informed consent. Anonymity was preserved using coded identifiers (T1–T6), which were used in reporting quotations. Participant demographic information is summarized in Table 1. In this study, Class Level A and Class Level B represent two student groups within the university’s English curriculum. Level A consists mainly of pure art students, while Level B includes students from other specialisations. These groups differ in learning dispositions and English proficiency, which shaped teachers’ experiences of implementing flipped classrooms.

**Table 1:** Demographic information of participants.

Teachers	Gender	Age	Years of teaching experience	Class level	Participated in flipped classroom program
T1	Male	35	9	A	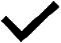
T2	Male	33	7	B	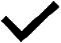
T3	Female	36	10	A	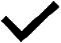
T4	Female	34	8	A	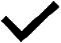
T5	Female	32	6	B	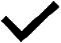
T6	Female	31	5	B	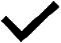

Data were collected in two phases: classroom observations and semi-structured interviews. Observations were conducted in the third week of the fall 2023 semester. A total of eight class session were observed. The researcher acted as a non-participating observer, taking detailed field notes on the classroom environment, student-teacher interactions, and teaching practices to supplement interview data and reduce self-report bias (Yin, 2014).

Following the observations, one-on-one semi-structured interviews were conducted via Tencent Meeting, each lasting 30–45 min. Open-ended questions encouraged participants to reflect on their experiences and strategies. An interview protocol was followed to ensure consistency, and all questions were pilot tested for clarity (see Table 2 for detailed interview questions). Interviews were recorded with consent and transcribed for analysis.

**Table 2:** Interview questions.

Questions
1. How has the flipped classroom model changed the way you plan and deliver your English lessons?
2. What kinds of mental or cognitive challenges have you encountered while preparing or conducting flipped classes?
3. How has your motivation for teaching changed—if at all—since adopting the flipped classroom method?
4. What factors have made it harder for you to stay motivated when using the flipped approach?
5. Based on your experience, what would you say are the most rewarding aspect and the biggest barrier to using the flipped model effectively in this university context?

**Figure 1:**
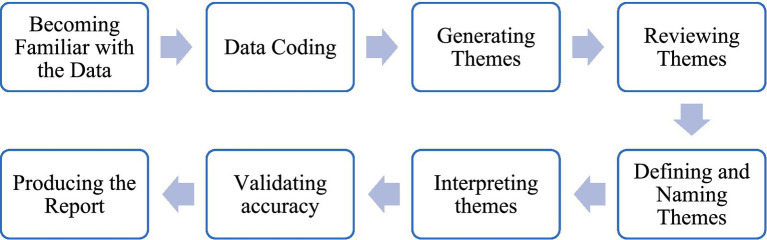
Data analysis process.

Thematic analysis is used as the method of data analysis in this research. This method involves the identification, organization, description, analysis, and reporting of themes present in a given data set. It is defined as short and simple to learn, but it is nonetheless an effective method for assessing the experiences of research participants (Braun and Clarke, 2014). Codes were generated inductively, grouped into categories, and refined into overarching themes representing shared experiences across participants. The data analysis process is presented in Figure 1.

Data analysis began with repeated readings of transcripts and field notes. *In vivo* coding was used to capture participants’ original language (Manning, 2017). Initial manual coding was followed by analysis in MAXQDA to identify recurring patterns and generate themes. A thematic map helped connect findings to the research questions. Bilingual quotes were presented where relevant, and translations were verified by a professional translator to ensure accuracy.

Trustworthiness in qualitative research was ensured through credibility, transferability, dependability, and confirmability (Lincoln and Guba, 1985). Credibility was enhanced through methodological triangulation—classroom observations, interviews, and focus groups (Yin, 2014)—and member checking, which allowed participants to validate transcripts and interpretations (Patton, 2015). An academic expert served as an interrater, with a coding agreement of 87%, exceeding the 80% benchmark for reliability. Transferability was supported through thick descriptions and purposive sampling from a specialized art-focused institution. Dependability was strengthened through detailed protocols, external review of the coding process, and re-analysis after a time lapse. A pilot study also improved consistency. Confirmability was established by grounding all interpretations in the data and maintaining transparency throughout, ensuring the overall rigor of the study.

## 4 Findings

This section presents the key findings from interviews with English teachers at a Chinese art university, highlighting their perceptions of the cognitive and motivational impacts of flipped classrooms on their teaching, as well as the challenges they faced during implementation.

### 4.1 Cognitive and motivational impacts on teaching

**Figure 2:**
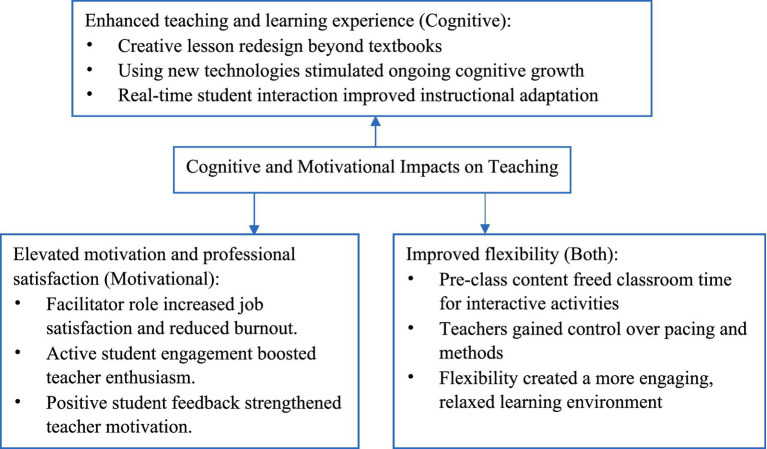
Cognitive and motivational impacts.

The analysis revealed three major themes reflecting how teachers perceived the flipped classroom approach to impact their teaching: enhanced teaching and learning experience, elevated motivation and professional satisfaction, and improved flexibility in instruction. A visual representation of these themes is presented in Figure 2.

Teachers reported that flipped teaching prompted them to rethink their instructional design and adopt more student-centered approaches. This shift encouraged deeper cognitive engagement in lesson planning and classroom facilitation, which ultimately enhanced their teaching and learning experience (see Extract 4.1 for details).

Extract 4.1 T3: “In the past, I just followed the textbook. Now I need to think *more creatively* about how to connect pre-class materials with in-class tasks. I have to learn *new technologies and new pedagogies*, which has been great for me.”T5: “The different technologies *influenced me a lot*, I *get excited* to learn and experiment with it which I couldn't do before.”T2: “Exciting as it is, I *felt much more*, the professional training *changed my teaching philosophy*. I feel more engaged as a teacher. I’m not just lecturing anymore; I’m responding to students’ reactions in real time, which pushes me to *keep learning and try to come up with good teaching plan*”

As can be seen in Extract 4.1, teachers reported that the flipped model encouraged them to move beyond textbook-based instruction and rethink their approach to lesson planning and delivery. T3, T5, and T2 described how engaging with flipped teaching pushed them to explore new technologies and pedagogical strategies, fostering professional growth and reshaping their instructional philosophies. These reflections indicate that the flipped approach stimulated cognitive engagement by requiring teachers to continuously adapt and innovate. The professional training accompanying the implementation introduced them to new tools and ideas, which many found intellectually stimulating and professionally rewarding.

Additionally, teachers consistently noted that using the flipped classroom model increased their motivation and professional satisfaction. They found it rewarding to see students more actively involved and appreciated the dynamic nature of flipped instruction (see Extract 4.2 for details).

Extract 4.2 T1: “My role is *completely different*. I was the centre of the class, and they all looked at me throughout the lesson. But now I spend most of my time *interacting* with them, 帮助他们成为更好的思考者, 对话者 (helping them to become *better thinkers and speakers*). I am *happy* that I am not the one doing all the talking.”T4: “I *don't want to go back* to old-school teaching. I might quit teaching because giving a lecture every day is *killing me*, especially when you are lecturing, and the students are sleeping. 翻转课堂让我变成了一个支持者(I have reshaped myself into a *supporter, facilitator in flipped classroom*). The focus is more on *offering help* instead of describing a topic very well.”T5: “I go around and ask if they have any questions, and it *feels good* to coach them *rather than do the thinking by myself*.”T6: “Freeing up the lecture time so that the teacher can engage with students, see how they think, clarify their understanding *brings satisfaction*.”T2: “I got one student calling me *'Captain'* as in the movie *Dead Poet Society*. I felt so *happy*, and I made up my mind that I will *keep making a difference*.”

As can be seen from Extract 4.2, teachers consistently described a noticeable increase in motivation and professional satisfaction after adopting the flipped classroom model. Many spoke of how the shift from traditional lecturing to student-centered facilitation helped them reconnect with the purpose and joy of teaching.

For T2, being called “Captain” by a student—referencing *Dead Poet Society*—symbolized a renewed sense of purpose and inspired him to continue innovating in his teaching. T4 shared that traditional lecturing left her emotionally drained and that flipped teaching revitalized her role, allowing her to become a supportive facilitator rather than just a content deliverer. Similarly, T1 and T5 found it rewarding to guide students’ thinking during class, while T6 appreciated the chance to engage directly with students’ understanding. Collectively, these reflections highlight how the flipped model reduced burnout, enhanced motivation, and brought greater professional fulfillment by making teaching more interactive and meaningful.

Moreover, teachers valued the flexibility that flipped teaching gave them in managing class time and structuring learning activities. By shifting content delivery to before class, they were able to use classroom time more effectively and adapt their teaching methods (see Extract 4.3 for details).

Extract 4.3 T6: “Pre-class video lectures allowed me to *free up time* to *explore* other teaching methods.”T3: “I *don't have to rush* to the class now, there is *more flexibility* for our teaching and students’ learning.”T1: “Now I have *more time in class* to *respond to students’ needs* rather than rushing through the textbook. I can also *try different activities*, *adapt more quickly*, and focus on communication skills rather than just grammar.”

As can be seen from Extract 4.3, teachers appreciated the flexibility that the flipped classroom model brought to their instructional routines. Shifting content delivery to pre-class videos enabled them to reclaim valuable classroom time and tailor in-class activities more effectively. For T6, using video lectures before class created space for experimenting with new approaches, allowing for more engaging and varied instruction. T3 emphasized the broader flexibility the model introduced for both teachers and students, noting a more relaxed and adaptable classroom rhythm. Similarly, T1 highlighted how flipped classroom enabled her to respond more effectively to students’ immediate learning needs, implement interactive activities, and shift the focus from textbook-driven grammar instruction to real-time communication skills.

### 4.2 Cognitive and motivational challenges on teaching

**Figure 3:**
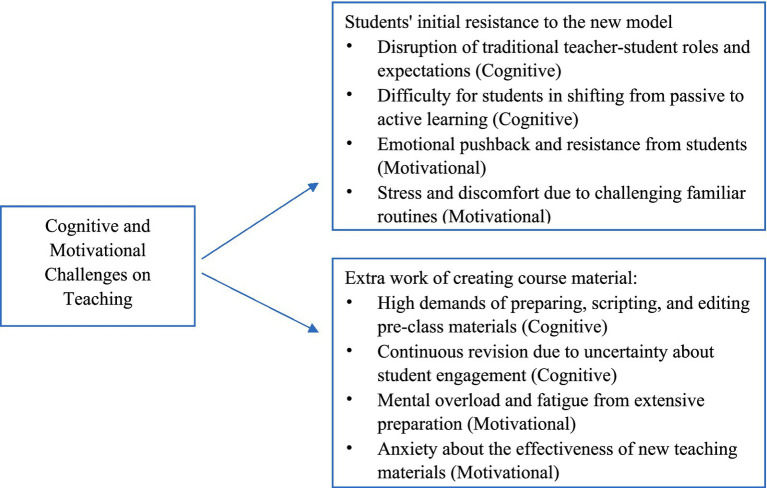
Cognitive and motivational challenges.

Teachers acknowledged that while the flipped classroom brought pedagogical benefits, its implementation also posed significant challenges. The analysis revealed two themes reflecting the cognitive and motivational challenges teachers perceived: students’ initial resistance to the new model and the extra work of creating course material. A visual representation of these themes is presented in Figure 3.

Many teachers reported resistance from students during the early stages of implementing the flipped classroom. While the model was designed to promote autonomy and active engagement, students’ expectations, habits, and previous learning experiences often clashed with the demands of the new approach (see Extract 4.4 for details).

Extract 4.4 T3: “I introduced the flipped classroom thinking it would be a 丝滑的过渡 (smooth transition), but the students *didn’t take it well*… 感觉自己触碰了一根红线, 起初确实存在反抗 (It felt like I had crossed some invisible red line—they *pushed back hard*)….”T6: “Faculty don't give students hard times, and *students give faculty good evaluations*. When it started to change, *students started to question* this familiar routine.”T1: "One of the biggest challenges was getting student…掌控自己的学习(take ownership of their learning). 他们太习惯做一个被动的学习者, 死记硬背 (They were *used to being passive listening*, taking notes, memorising). With the flipped classroom, they suddenly had to prepare before class and actively participate. 不仅对他们, 对我来说都是一个思维的转变 (It wasn’t just a change for them; it was also a mindset shift for me as a teacher). We both had old habits. 熟悉的角色不再适用了, 一开始这对所有人来说都不舒服 (The *traditional roles didn’t apply anymore*, and that was uncomfortable at first—for everyone)."

As can be seen from Extract 4.4, student resistance was a major hurdle for many teachers when introducing the flipped classroom model. T3 reflected on how the transition, which he initially anticipated would be smooth, instead triggered strong pushback. His metaphor of crossing a “red line” suggests that the change disrupted deeply ingrained expectations about classroom roles and norms. T6 further explained that an unspoken agreement often exists in traditional settings—teachers avoid placing excessive demands on students, and in return, students provide compliance and favorable evaluations. When the flipped model disrupted this tacit understanding by increasing student responsibility, it was met with skepticism and discomfort. T1 captured the core of the issue by highlighting students’ long-standing passivity. He noted that learners were accustomed to memorizing rather than engaging critically, and that asking them to take initiative represented a significant mindset shift. Importantly, T1 also acknowledged that the shift was challenging not only for students but also for himself as a teacher. The familiar dynamics and roles no longer applied, leading to initial discomfort for all involved.

In addition, the extra workload of creating also poses challenges. Although they acknowledged the long-term benefits of flipped teaching, the initial preparation phase was often described as overwhelming and cognitively demanding (see Extract 4.5 for details).

Extract 4.5 T5: “我低估了自己的工作量 (I underestimated how much explanation I would need to provide). I ended up spending hours just preparing a single lesson.”T4: “The training inspired me, but the *application was another story*. The *mental load was heavier* than I thought. Designing everything from scratch—videos, tasks, and class discussions—really challenged my time and energy.”T2: “I kept *reworking* my materials because I wasn’t sure how students would respond. Every week felt like a gamble. The pre-class part wasn’t just recording a video—it was scripting, editing, uploading, and then thinking about what to do in class.”

As illustrated in Extract 4.5, flipped teaching required a considerable investment of time and cognitive effort from teachers, particularly during the early stages of implementation. T5 admitted to underestimating the workload involved, especially the effort needed to introduce and justify the new model to students. Similarly, T2 highlighted the gap between theoretical enthusiasm and the practical demands of application. The need to create new materials, align them with classroom activities, and anticipate student reactions resulted in what he described as a heavy mental burden. T6’s account reinforces this experience, detailing the iterative nature of preparation—teachers often had to refine their materials repeatedly due to uncertainty about student engagement. Her description also reveals the multifaceted nature of pre-class work, involving not just content creation but also technical and pedagogical planning.

## 5 Discussion

This discussion interprets the findings through the lenses of Social Constructivist Theory (Vygotsky, 1978) and self-determination theory (Deci and Ryan, 1985), examining how flipped classroom implementation shaped teachers’ cognitive and motivational experiences within the specific context of Chinese art universities.

### 5.1 Cognitive engagement and constructivist learning theory

The flipped classroom model observed in this study demonstrates a clear alignment with Constructivist Learning Theory proposed by Vygotsky (1978). According to the theory, learning is not passively received but actively constructed by learners through interaction with their environment and others (Vygotsky, 1978). While originally developed to describe student learning, this theory also provides a valuable lens for understanding teachers’ pedagogical development, particularly in active and reflective teaching contexts.

Teachers in the current study reported a significant shift away from textbook-driven delivery toward more interactive, student-centered lesson planning. This pedagogical transformation required greater cognitive engagement, as teachers became instructional designers, facilitators, and reflective practitioners. Such a transition illustrates the teacher-as-learner dimension of constructivism, where teachers construct new knowledge about teaching through practice, experimentation, and reflection. Additionally, the requirement to integrate new technologies, design multimodal resources, and align these with classroom tasks in flipped classroom echoes the constructivist emphasis on contextualized, authentic learning.

Teachers were not merely transferring knowledge; they were curating experiences that enabled knowledge construction, often with real-time feedback and mutual meaning-making. In this way, the flipped model extends constructivist principles from the student domain into teacher cognition and practice. This supports Erbil’s (2020) view of the flipped classroom as a social constructivist model that promotes cooperative learning and peer interaction. Similarly, Lo et al. (2017) observed that flipped instruction fosters reflective teaching and responsiveness to student needs.

### 5.2 Enhanced teacher motivation through the lens of self-determination theory

Self-determination theory (SDT) provides a valuable lens for understanding the motivational shifts experienced by teachers in this study. According to SDT, human motivation is influenced by the fulfillment of three basic psychological needs: autonomy, competence, and relatedness (Deci and Ryan, 1985). The flipped classroom environment, as described by participants, appeared to support these needs and thereby enhanced intrinsic motivation and professional engagement.

The model offered greater autonomy in instructional design. Teachers reported that they could make more creative and independent decisions regarding how and what to teach—moving away from rigid textbook structures toward designing interactive and student-responsive activities. This freedom in pedagogical decision-making corresponds closely with the SDT principle that autonomy enhances self-motivation and job satisfaction. Similar findings have been reported in recent flipped classroom studies, where the shift toward teacher-led content design was shown to strengthen pedagogical agency and innovation (Hew et al. 2021; Baig and Yadegaridehkordi, 2023). In this study, autonomy was not only structural but also psychological, as teachers reported feeling empowered to make meaningful changes to their teaching style.

Many teachers expressed a renewed sense of competence. As they developed new skills in educational technology, experimented with multimodal materials, and successfully adapted their lessons to the flipped format, they experienced a sense of professional growth and mastery. For example, teachers described becoming more confident facilitators, responding in real time to student needs, and enjoying the challenge of designing dynamic learning environments. Such feelings align with what SDT identifies as the importance of perceived competence in fostering intrinsic motivation (Deci et al., 2017). Such feelings align with what SDT identifies as the importance of perceived competence in fostering intrinsic motivation (Deci et al., 2017). This is consistent with findings by Chen Hsieh et al. (2017) who also found that flipped teaching increased teacher confidence and technical competence through iterative practice and reflection.

The flipped classroom allowed teachers to build stronger relatedness with students. By spending less time lecturing and more time interacting, guiding, and responding to learners during class, many teachers felt a deeper connection with their students. For instance, one teacher highlighted being called “Captain” by a student, which symbolized emotional recognition and mutual respect. These kinds of interactions reflect the SDT notion of relatedness: the human need to feel connected to others in meaningful ways. Recent studies have emphasized that flipped learning environments promote emotional presence and relational pedagogy, particularly by restructuring classroom interactions around mutual dialogue and feedback (Li and Yang, 2022; Zhang and Hu, 2023). In this study, moments of shared recognition and engagement helped redefine teacher–student relationships in more personally meaningful terms.

Beyond these individual dimensions, the findings indicate that teachers’ motivational needs were not experienced in isolation but fluctuated dynamically across different phases of flipped classroom implementation. During the preparation stage, teachers experienced heightened autonomy as they redesigned materials and crafted pre-class activities, though this was tempered by the additional workload of content creation. In the facilitation stage, competence needs became most salient, as teachers had to manage classroom dynamics and overcome initial student resistance. Relatedness was emphasized most strongly in post-class reflection, where feedback and interaction with students and colleagues reinforced professional satisfaction. These needs also interacted: autonomy in design provided the foundation for exercising competence in delivery, while relatedness through feedback and recognition sustained teachers’ motivation to persist with innovation. This fluctuation illustrates that motivational processes during pedagogical change are complex and interdependent, shaped not only by psychological needs but also by cognitive pressures and institutional contexts.

### 5.3 Teacher challenges as reflections of learner realities in the Chinese art university context

The cognitive and motivational challenges faced by teachers in this study were not experienced in isolation but were deeply intertwined with the characteristics of the learners and the institutional context of Chinese art universities. Specifically, student resistance to the flipped classroom model and the increased cognitive demands of instructional preparation both reflected underlying systemic and institutional learning conditions that shaped teaching and learning in this setting.

Firstly, student resistance emerged as a cognitive challenge for teachers, reflecting a mismatch between the flipped classroom’s learner-centered philosophy and students’ entrenched expectations of teacher-led, exam-oriented instruction. As shown in the findings, several teachers noted that students were unprepared or unwilling to take ownership of their learning, having been habituated to passive modes of instruction focused on memorization. For instance, T1 remarked that “They were used to being passive learners,” indicating how flipped learning disrupted familiar roles. This is consistent with prior research in Chinese higher education, where students in non-academic disciplines such as arts have been observed to lack independent learning strategies and exhibit lower academic motivation (Zhang, 2012; Liang, 2015). Comparatively, studies in more general higher education settings have found that flipped classrooms are sometimes met with similar initial resistance, though the severity and nature of challenges vary depending on institutional culture, student preparedness, and discipline (Kong et al., 2024). The discomfort reported by both teachers and students in this study highlights how cognitive demands—such as the need to rethink learning roles and engage in self-regulated preparation—were intensified by contextual norms.

In parallel, teachers experienced motivational challenges in the form of heightened workload and mental fatigue due to the necessity of preparing and adapting flipped materials. As reflected in the finding from T4 and T2, who described the planning process as “heavier than expected” and “a gamble,” this burden was compounded by uncertainty about student engagement and inconsistent preparation. These concerns echo broader observations that many Chinese art universities face limitations in digital resources, training support, and institutional incentives for pedagogical innovation (Li, 2019). Teachers thus found themselves investing substantial effort into flipped materials without guarantees of student uptake or administrative recognition, which undermined motivational resilience. As Deci et al. (2017) point out, when perceived effort outweighs perceived efficacy or support, intrinsic motivation can decline—especially in environments that do not provide feedback or reinforcement.

Furthermore, the institutional learning culture within Chinese art universities may intensify these challenges. Compared to science or business faculties, students in art-focused institutions are often perceived to be more expressive yet less academically disciplined, and less accustomed to structured, high-effort learning outside class (Zhang, 2012). Teachers’ attempts to implement flipped learning therefore clashed not only with institutional traditions but also with prevailing student dispositions. T3’s reflection that student pushback felt like crossing a “red line” metaphorically captures the risk of deviating from unwritten expectations about classroom norms, which can lead to demotivation and feelings of professional vulnerability.

## 6 Conclusion

This study explored the cognitive and motivational impacts of flipped classrooms on English teachers in a Chinese art university, as well as the challenges they encountered during implementation. Drawing on social constructivist and self-determination theories, the findings revealed that the flipped model encouraged deeper cognitive engagement, enhanced professional motivation, and enabled more flexible, student-centered teaching practices. Teachers reported a renewed sense of autonomy, competence, and relatedness—core elements of intrinsic motivation—through their involvement in designing and facilitating flipped lessons.

However, the transition was not without difficulty. Teachers faced cognitive demands in preparing new materials and adapting pedagogical approaches, while also navigating students’ initial resistance rooted in passive learning norms and traditional expectations. These challenges were closely tied to contextual factors, including limited student preparedness and institutional constraints common in Chinese art university settings.

At the same time, the findings are shaped by the homogeneity of the sample: six teachers from the same institution and discipline, with similar levels of teaching experience and prior exposure to flipped pedagogy. This institutional and disciplinary specificity may have influenced how teachers perceived both the benefits and challenges of flipped classrooms. As such, while the study offers valuable contextualized insights, the findings are not intended to be directly generalizable. Instead, they highlight the importance of considering how institutional culture, teaching traditions, and disciplinary norms mediate teachers’ responses to pedagogical innovation.

In addition, the study relies exclusively on teacher perspectives, which provides rich insight into instructional strategies and motivational processes but does not capture students’ experiences or engagement. As such, while the study offers valuable contextualized insights, the findings are not intended to be directly generalizable. Instead, they highlight the importance of considering how institutional culture, teaching traditions, and disciplinary norms mediate teachers’ responses to pedagogical innovation, and they point to the potential value of incorporating student feedback in future research to gain a more holistic understanding of flipped classroom dynamics.

## 7 Recommendation for future study

Future research might extend these insights and investigate:

- The long-term effects of flipped classrooms on teacher identity, cognition, and instructional practice over time.
- Students’ cognitive and motivational experiences in flipped classrooms to complement teacher-focused findings and provide a more comprehensive view.
- Different types of Chinese higher education institutions to understand how institutional context influences flipped classroom implementation.

## Data Availability

The original contributions presented in the study are included in the article/supplementary material, further inquiries can be directed to the corresponding author.
